# Molecular Imaging in Genetic Medicine

**DOI:** 10.7759/cureus.565

**Published:** 2016-04-11

**Authors:** Faiq Shaikh, Ayden Jacob, Frederick Van Gestel, Shahriar Yaghoubi

**Affiliations:** 1 Imaging Informatics, University of Pittsburgh Medical Center, Pittsburgh, PA.; 2 Molecular Imaging, Cellsight Technologies, Inc., San Francisco, CA.; 3 Director of Translational Medicine, Nanoaxis LLC, Neuroscientist, Neuro-Nanotech Division, University of California, Department of Bioengineering; 4 UCSF Department of Interventional Radiology and Oncology; 5 2nd Master Medicine, Catholic University of Leuven; 6 Medical Affairs, United Therapeutics

**Keywords:** molecular, imaging, genetic, reporter gene, medicine, optical

## Abstract

The field of biomedical imaging has made significant advances in recent times. This includes extremely high-resolution anatomic imaging and functional imaging of physiologic and pathologic processes as well as novel modalities in optical imaging to evaluate molecular features within the cellular environment. The latter has made it possible to image phenotypic markers of various genotypes that are implicated in human development, behavior, and disease. This article discusses the role of molecular imaging in genetic and precision medicine.

## Introduction and background

Reporter genes (RGs) are responsible for encoding proteins that can be rapidly and sensitively assayed as surrogate markers when fused with regulatory regions of the gene of interest [1]. They are used to study promoter/enhancer elements that affect expression and messenger ribonucleic acid (mRNA) sequences that control message stability and regulate translation efficiency [1-2]. That is made possible in living subjects through the development of highly advanced molecular imaging techniques within the domain of optical imaging [2]. RGs are increasingly being used for imaging-based evaluation and monitoring of gene and cell therapy, stem cell therapy, and immune modulation therapy as they provide the means to determine the location, magnitude, and persistence of gene expression and monitor cell kinetics (cell biodistribution, quantity, proliferation, survival, differentiation, and function) in living organisms [3-4].

## Review

Reporter gene (RG) imaging involves non-invasive, repetitive, and sometimes quantitative analysis of reporter gene expression [5]. The process evolves from RG transcription into enzyme/receptor/transporter production, leading to entrapment of the imaging reporter probe, which then imparts the signal for imaging. This may be a radioisotope/photochemical reaction/magnetic resonance metal cation based on the imaging modality used [1-2]. Currently available non-RG-based molecular imaging techniques depend on the direct interaction of imaging probes with their cellular targets, causing retention of imaging probe within tissues, such as radiolabeled ligands and radiolabeled antibodies. One such example is that of 11C-WAY-100635 (radioligand for the 5-HT1A receptor) or iodine-124 radiolabeled anti-carcinoembryonic antigen minibody [6].

**Figure 1:**
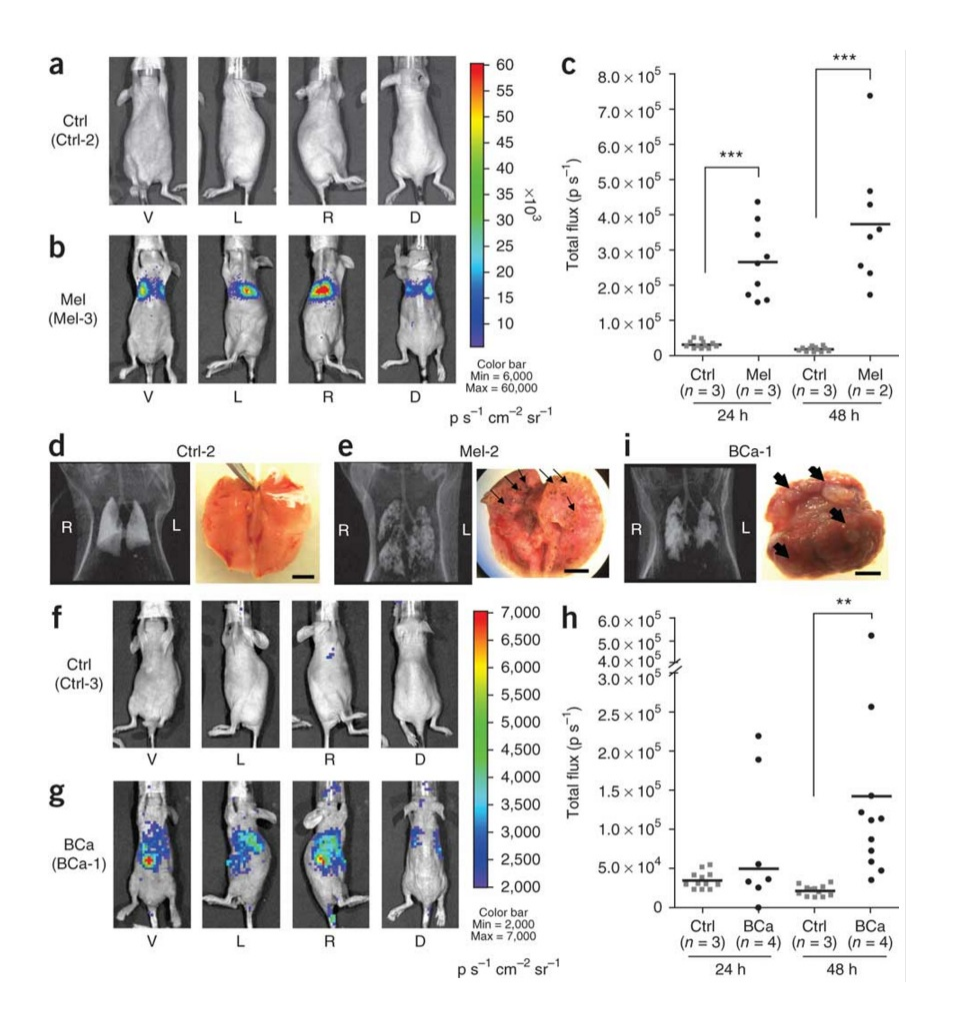
Bioluminescence imaging in cancer models Bioluminescence imaging using firefly luciferase expression in a (a) control mouse model, (b) mouse melanoma model at 24 and 48 hrs after injection of pPEG-Luc–PEI polyplex, (c) flux graph, along with their respective (d-e) gross specimen photographs and CT images; bioluminescence imaging using firefly luciferase expression in a (f) control mouse model, (g) mouse breast cancer model at 24 and 48 hrs after injection of pPEG-Luc–PEI polyplex, (i) flux graph, along with their respective (i) gross specimen photo and CT scan. (Used with permission from Pomper, et al.: Tumor-specific imaging through progression elevated gene-3 promoter-driven gene expression. Nature Medicine 2011 17:123–129)

RG imaging provides a non-invasive and highly specific means of detecting several molecular processes, such as gene expression or protein-protein interactions. This enables us to optimize drug and gene therapy, assess disease progression at a molecular level, and allow rapid, reproducible, and quantitative monitoring of time-dependent influences on gene products [3, 7]. Optical imaging involves techniques, such as fluorescence and bioluminescence, to detect visible light that is generated in living cells. Fluorescence imaging uses proteins that emit photons as a result of excitation with those of a shorter wavelength and can be used to microscopically track the subcellular distribution of various molecules and monitor expression of gene products. Bioluminescence depends on the enzymatic reaction between a luciferase enzyme (firefly, Renilla) and its substrate (D-luciferin, coelenterazine) to produce visible light that is detected and quantified using highly sensitive cooled charge-coupled device (CCD) cameras (Figure 1) [8-9].

There have been significant advances in magnetic resonance imaging (MRI) and ultrasonography-based imaging of subcellular processes. MRI-based probes, such as β-gal + activated contrast agent, ferritin, tyrosinase, magA, plasma membrane-bound peptides, and engineered transferrin receptors (ETfR) have been introduced to monitor gene expression patterns in gene therapy and to analyze processes of cell-based regenerative therapies within oncology, cardiology, and neurology [10]. Acoustically analyzable perfluorocarbon nanoparticles have been used to detect speciﬁc receptors as well [11].

**Figure 2:**
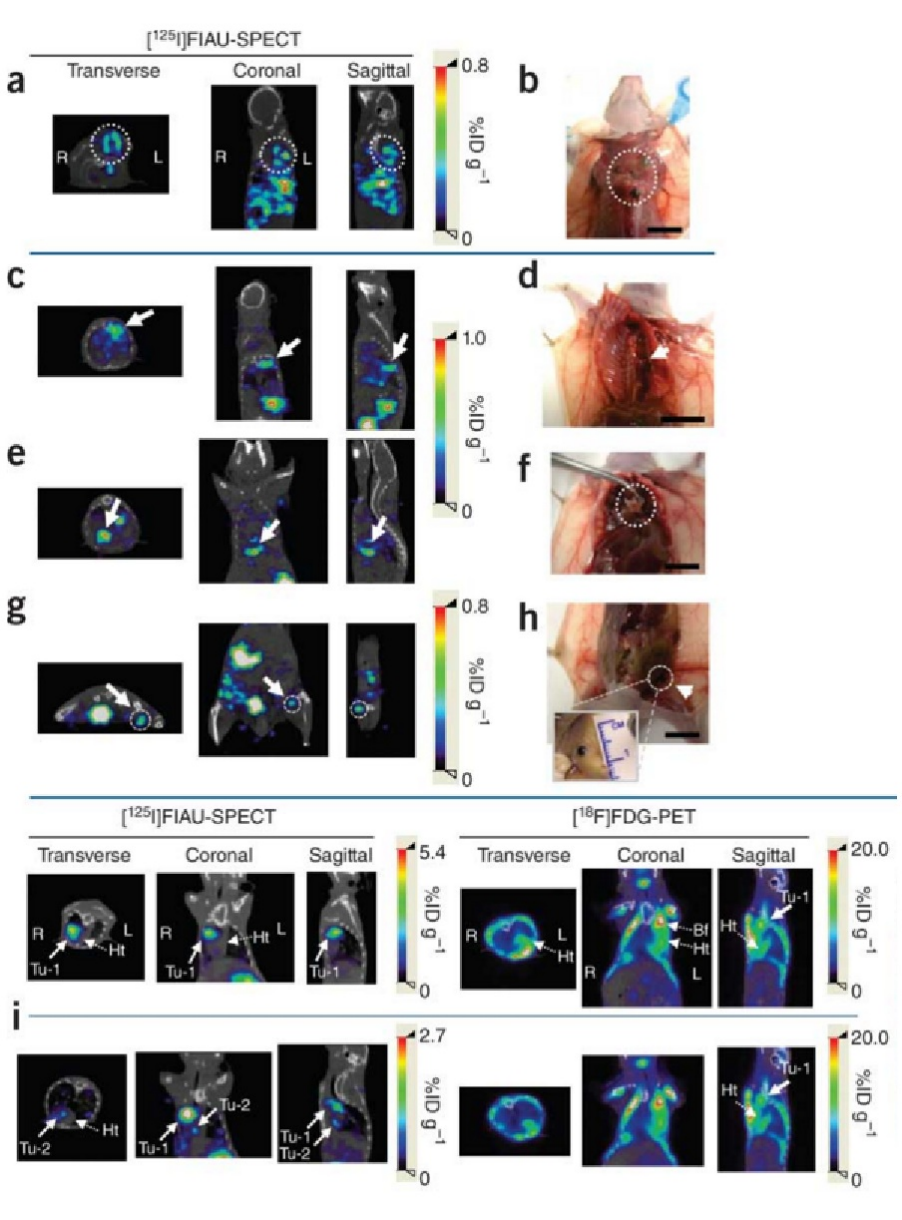
125I-FIAU SPECT vs. 18F-FDG PET imaging Comparison of PEG-3 promoter mediated 125I-FIAU SPECT (with corresponding specimen photographs; a-h) and (i) 18F-FDG PET imaging in a mouse breast cancer model. FIAU identified two tumor sites obscured by physiologic distribution of FDG on PET imaging. (Used with permission from Pomper, et al.: Tumor-specific imaging through progression elevated gene-3 promoter-driven gene expression. Nature Medicine 2011 17:123–129)

**Figure 3:**
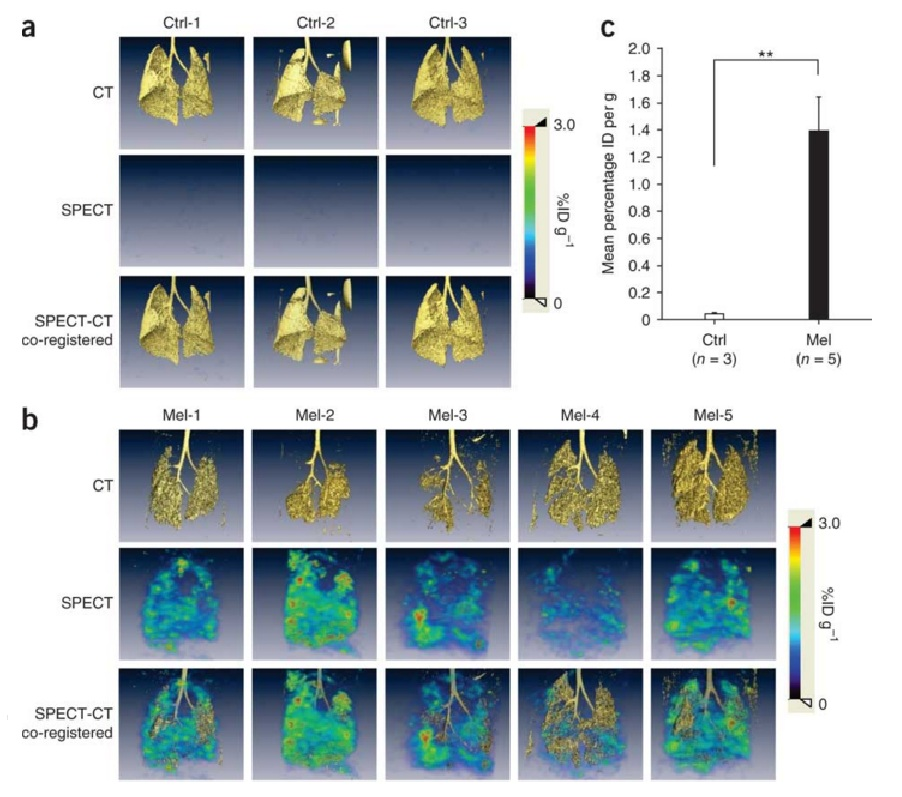
Pulmonary metastasis identified by 125I-FIAU SPECT/CT imaging CT, SPECT, and fused SPECT/CT 125I-FIAU imaging after injection of pPEG-HSVtk-PEI in (a) control vs. (b) mouse melanoma model. (Used with permission from Pomper, et al.: Tumor-specific imaging through progression elevated gene-3 promoter-driven gene expression. Nature Medicine 2011 17:123–129)

In the field of Nuclear Medicine, radioisotopes that emit high energy particles like gamma rays and positrons to label probes are used, which readily penetrate tissue and can be detected outside the body. Gamma- or positron-emitting isotopes can be incorporated into molecular probes, such as receptor-speciﬁc ligands and enzyme-speciﬁc substrates. Imaging agents, such as 1-(2-deoxy-2-fluoro-1-D-arabinofuranosyl)-5-iodouracil (FIAU), have been used to image the expression of their respective RGs using positron emission tomography (PET) or single photon emission computed tomography (SPECT), depending on the labelled radiotracer (iodine-124 for PET; iodine-123 or -125 for SPECT) (Figures 2-3). These RGs may encode enzymes that phosphorylate specific PET/SPECT reporter probes that lead to their intracellular entrapment, or encode receptors that can bind to specific probes, or encode cell membrane transporters that facilitate flow and accumulation of these specific probes into cells [12-13]. 

PET/SPECT reporter probes can be either human gene-based or virus/bacteria-based. Herpes simplex virus Type 1 thymidine kinase (HSV1-tk) and its mutant derivatives are the most extensively studied and applied viral-based reporter probes. Immunogenicity of viral-based probes can be detrimental to adoptively transferred therapeutic cells expressing them, shortening the duration of their expression, or eradicating the therapeutic cell expressing them [14]. Conversely, human gene-based IRGs may accumulate in cells that express the endogenous gene, or may mimic as the endogenous gene, causing perturbation in the functioning of the cells in which it is expressed. Human thymidine kinase 2 (hmtk2) is an example of a human gene product which is ubiquitously expressed in the mitochondria of mammalian cells. It is inaccessible to uracil nucleoside analogs and has a non-immunogenic truncated version produced in the cytoplasm without nonspecific accumulation of its probes [2, 15]. 

**Figure 4:**
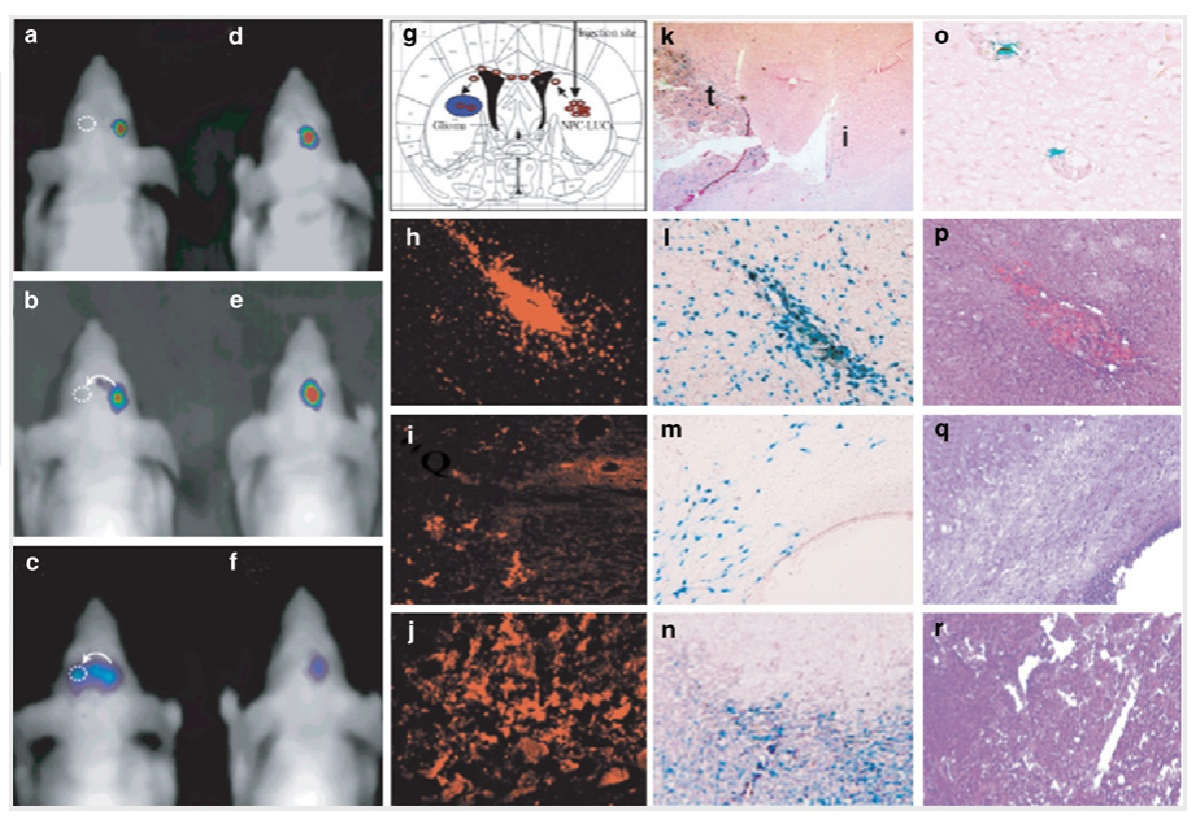
Bioluminescence imaging demonstrating parenchymal migration of luciferase-expressing cells Parenchymal migration of NPC-LUCs expressing Fluc (NFC-Luc) cell that was implanted into (a-c) the right hemisphere of either mice bearing Gli36 tumors in the left hemisphere or (d-f) control mice which did not have tumors. Time intervals are represented at (a) Day 0, (b) 1 week, and (c) 2 weeks. Migration towards the tumor (dotted circle) was first noted after 1 week and migration across the midline was evident at 2 weeks. Images d-f are from the control, along with (g) corresponding schematic image, and (h-j) histographs with anti-luciferase staining, (k-n) X-gal staining, and (o-r) H&E staining. (Used with permission from Shah, et al.: Molecular imaging of gene therapy for cancer. Gene Therapy 2004 11:1175–1187)

**Figure 5:**
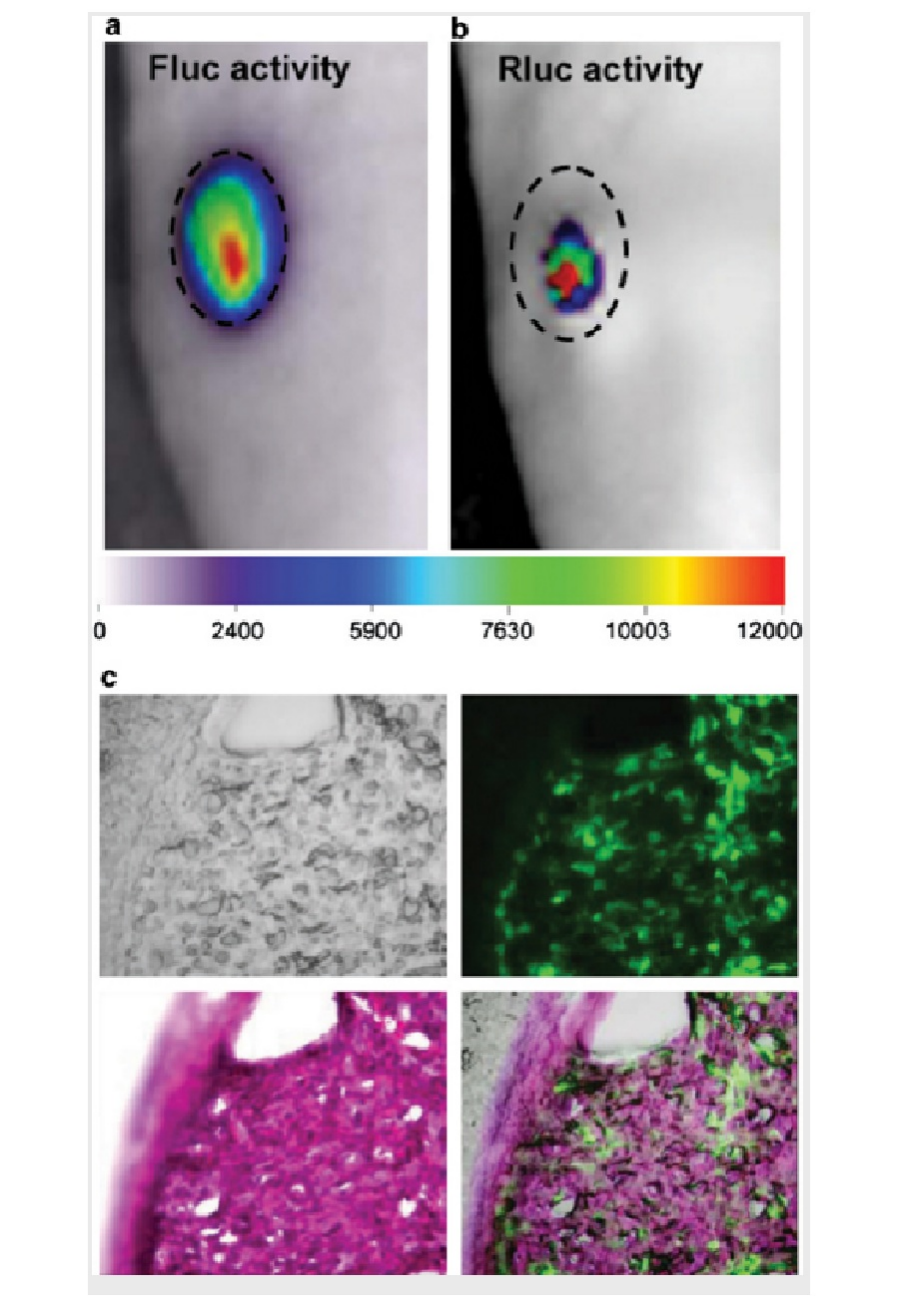
Bioluminescence dual imaging of amplicon vector delivery and glioma volumes (a) Mouse model bearing subcutaneous Gli36fluc+ gliomas were injected i.p. with D-luciferin and imaged for Fluc activity. (b) A-Rluc amplicon vector injected into the same tumor and 36 h later, coelenterazine was injected into the tail vein and the mice were imaged for Rluc activity. The dashed circle around the tumor indicates the tumor periphery. (c) Histopathology showing tumor cells in the tissue sections, GFP-positive cells in the tumor; and H&E stained tumor. (Used with permission from Shah, et al.: Molecular imaging of gene therapy for cancer. Gene Therapy 2004 11:1175–1187)

Molecular imaging techniques also provide ways to study the whole-body kinetics of therapeutic cells (TCs), allowing for monitoring presence, location, quantity, proliferation, survival, and status of TCs in animals or patients at any desired time-point following their administration, such as seen in luciferase-based imaging here (Figures 4-5). Whole-body 18F-FHBG PET scans have been performed to demonstrate above-background signal at the site of cytolytic T-cell infusions, which revealed trafficking of these cells to a remote recurrent tumor in the patient's corpus callosum [16]. 

The next main application of imaging genetics in medicine is to monitor gene therapy for bioavailability and to determine biodistribution and kinetic profiles. This will entail modalities that image deeper tissue, such as optoacoustics, ultrasound, MRI, and PET. The first gene therapy trial for the treatment of severe combined immunodeficiencies using the gene for adenosine deaminase took place in 1990 [17]. Since then, there has been a significant advancement in gene therapeutics, such as the introduction of an aerosol cystic fibrosis transporter gene [18], subretinal FLT1 therapy for age-related macular degeneration [19], GUC2YD gene therapy for Leber’s amaurosis [20], intramuscular Factor IX therapy for hemophilia B [21], and intramuscular AAT gene therapy for hereditary emphysema [20], etc. Report gene imaging may also have a role in monitoring T-cell receptor expression in leukocytes, which is the key mechanism of gene-based immunomodulators used for cancer therapy [22].

## Conclusions

Overall, reporter gene imaging is beginning to play an important role in expanding our understanding of disease mechanisms and in optimizing and developing new gene- and immunotherapy. As these novel approaches become clinically applied, notably the chimeric antigen receptor T-cell (CAR-T) therapy, the role of RG imaging appears to hold great promise as the driving force for genetic/precision medicine.
